# PSYCHOMETRIC PROPERTIES OF THE KOREAN VERSION HOSPITAL SURVEY ON PATIENT SAFETY CULTURE IN HOME HEALTHCARE

**DOI:** 10.1093/geroni/igad104.3540

**Published:** 2023-12-21

**Authors:** Kayoung Lee, Soohee Lee, Seoyoon Woo

**Affiliations:** Gachon University, Incheon, Gyeonggi, Republic of Korea; Gachon University, Incheon, Gyeonggi, Republic of Korea; University of North Carolina Wilmington, Wilmington, North Carolina, United States

## Abstract

Patient safety culture is essential for quality care in healthcare organizations. Instruments for patient safety have been developed and widely implemented in hospital settings. The revised hospital survey on patient safety culture (HSOPSC) is one of the representative instruments for evaluating patient safety culture. In 2021, the Korean version of the HSOPSC 2.0 (K-HSOPSC 2.0) was developed. However, there is a lack of evidence to examine the validity of the instrument in home healthcare settings. This study aimed to test the psychometric analysis of K-HSOPSC 2.0 in home healthcare settings in South Korea. We collected 215 certified advanced practice nurses (APNs) and nurse managers from 163 out of 175 home healthcare institutions in South Korea. We conducted the confirmation factor analysis (CFA) to examine the existing 10 dimensions of K-HSOPSC 2.0. Statistical analyses were performed with Stata 17.0, and the Satorra-Bentler (SB) method was used to adjust the standard error. The construct validity demonstrated an acceptable fit (RMSEA_SB=0.061, CFI_SB=0.912, TLI_SB=0.889). The K-HSOPSC 2.0 demonstrated high reliability (Cronbach’s α=0.90), except for teamwork (α=0.52) and staffing and work pace (α=0.39) among 10 dimensions. The validity of the measurement of patient safety culture was supported in home healthcare settings. However, further evaluation is needed for the two dimensions of ’teamwork’ and ’staffing and work pace’. In home healthcare settings, it is crucial to consider the role of patients and caregivers in teamwork and different home healthcare working environments in staffing that reflects unique home healthcare settings.

